# Is there an association between out-of-pocket hospital costs, quality and care outcomes? A systematic review of contemporary evidence

**DOI:** 10.1186/s12913-023-09941-3

**Published:** 2023-09-13

**Authors:** Ramya Walsan, Rebecca J. Mitchell, Jeffrey Braithwaite, Johanna Westbrook, Peter Hibbert, Virginia Mumford, Reema Harrison

**Affiliations:** 1 Centre for Health Systems and Safety Research, Australian Institute of Health Innovation, Faculty of Medicine and Health Sciences, Macquarie University, Level 6, 75 Talavera Road, Sydney, NSW 2109 Australia; 2 Centre for Healthcare Resilience and Implementation Science, Australian Institute of Health Innovation, Faculty of Medicine and Health Sciences, Macquarie University, Sydney, Australia

**Keywords:** Out of pocket costs, Gap payment, Patient payment, Healthcare quality, Health outcomes

## Abstract

**Background:**

Out of pocket (OOP) costs vary substantially by health condition, procedure, provider, and service location. Evidence of whether this variation is associated with indicators of healthcare quality and/or health outcomes is lacking.

**Methods:**

The current review aimed to explore whether higher OOP costs translate into better healthcare quality and outcomes for patients in inpatient settings. The review also aimed to identify the population and contextual-level determinants of inpatient out-of-pocket costs. A systematic electronic search of five databases: Scopus, Medline, Psych Info, CINAHL and Embase was conducted between January 2000 to October 2022. Study procedures and reporting complied with PRISMA guidelines. The protocol is available at PROSPERO (CRD42022320763).

**Findings:**

A total of nine studies were included in the final review. A variety of quality and health outcomes were examined in the included studies across a range of patient groups and specialities. The scant evidence available and substantial heterogeneity created challenges in establishing the nature of association between OOP costs and healthcare quality and outcomes. Nonetheless, the most consistent finding was no significant association between OOP cost and inpatient quality of care and outcomes.

**Interpretation:**

The review findings overall suggest no beneficial effect of higher OOP costs on inpatient quality of care and health outcomes. Further work is needed to elucidate the determinants of OOP hospital costs.

**Funding:**

This study was funded by Medibank Better Health Foundation.

**Supplementary Information:**

The online version contains supplementary material available at 10.1186/s12913-023-09941-3.

## Introduction

Rising healthcare costs, coupled with changing healthcare needs, have led to patients being increasingly required to either fully, or partially, cover the cost of their healthcare [1]. As a consequence, healthcare payments made by consumers in many countries, known as out of pocket (OOP) costs, have been rising steadily [2]. According to the World Health Organization (WHO), around 150 million people worldwide are faced with catastrophic health expenditures due to OOP costs and 100 million people are forced to live below the poverty line due to OOP costs [3]. In Australia, consumers pay around 17% of total health expenditure directly through OOP payments [4]. This represents an annual expenditure of some AUD $4,290 per household or 5.8% of all expenditure on household goods and services [5]. The impact of OOP costs is more pronounced in complex health care contexts, such as the United States (US), where more than quarter of the population report a health-related financial burden exceeding 20% of their family income [6]. Even in countries with compulsory social health insurance schemes such as Europe, around a fifth of all healthcare spending comes directly from patients through OOP [7, 8].

Rising OOP costs are recognised as a critical healthcare challenge. Several studies indicate that high OOP costs are associated with underutilisation of necessary healthcare services, lower treatment adherence, and increased hospitalisation and emergency department visits [9–11]. Forgone care due to OOP costs can in turn lead to poorer health and higher cost outcomes for patients, and can place additional burden on healthcare systems in the longer-term due to people not seeking timely care [2]. There is some evidence to suggest that the burden of OOP costs can be higher for individuals who experience health conditions, such as cancer, stroke or long-term chronic diseases due in part to their higher healthcare utilisation than by other population groups [11–13]. Unwarranted treatment cost variation, that is, marked variation in both total treatment costs and in OOP costs for the same treatment conducted at facilities in different geographical locations and by different health professionals, has also been reported in health systems internationally [14–16]. Less is known however about the relationship between OOP costs and the quality of healthcare. Proponents of OOP costs argue that OOP costs can act as a source of revenue, increase patient awareness of treatment costs and curb unnecessary service use [17, 18]. Increased resource availability could have a favourable effect on the quality of care [19]. In contrast, health facilities may be reluctant to modify their resource input and can prioritise profit maximisation despite charging more from patients [19]. Changes in OOP costs may be unrelated to quality of care provided in this instance.

The amount and nature of OOP payments varies considerably across countries and healthcare systems [18]. In general, low- and middle-income countries are reported to have higher shares of OOP payments compared to high-income countries where financial risk pooling mechanisms are well developed and government assumes a greater responsibility in paying healthcare costs [3]. The WHO defines OOP spending as catastrophic when it exceeds more than 40% of total household income [20]. It has been observed that individuals from lower income populations tend to incur higher healthcare spending in relation to their income and may settle for a lower quality of health care or forgo seeking healthcare altogether as a result, compared to those with higher income levels [21]. Countries have adopted different approaches to reduce OOP spending such as the introduction of universal insurance systems, abolishing user fees, or eliminating OOP payments for certain demographic groups and people on low income [22–24]. Nonetheless, high healthcare expenses still persist in high income countries that disproportionately affect individuals from socio economically disadvantaged backgrounds [23].

Given the degree of burden imposed by OOP costs on consumers, it is imperative to understand the evidence available on the association between OOP costs and quality of care, particularly in hospital settings that attract higher OOP costs. It will also be beneficial to identify which characteristics make patients more vulnerable to OOP payments. Accordingly, the purpose of this systematic review is to synthesise and report evidence of the association between OOP costs and healthcare quality and health outcomes in inpatient settings to inform healthcare policy and consumer decision making. Specifically, the review aimed to examine whether higher OOP costs translate into better healthcare quality and outcomes for patients, in addition to identifying the population and contextual level factors that may contribute to the level of OOP cost incurred. The following research questions were addressed: 1. To what extent is there an association between OOP costs and care quality and health outcomes? 2. What population and contextual factors are associated with higher OOP costs?

## Methods

This review is reported in accordance with the Preferred Reporting Items for Systematic Review and Meta-Analysis (PRISMA) [25]. The protocol for this review was registered on PROSPERO (CRD42022320763).

### Eligibility criteria

Studies were eligible for inclusion if they used an observational or experimental design to examine the effects of OOP costs on quality and health outcomes in inpatient settings. OOP costs were defined as payment for inpatient medical care that was not covered by universal health insurance, private insurance or any other similar sources [26]. OOP costs often take one of three forms: co-payments, deductibles, and coinsurance [27]. Co-payments are payments that an individual makes for medical services [28]. Deductibles are payments an individual makes for a covered medical service before their insurance company reimburses [29]. Coinsurance is the percentage of treatment costs an individual pays after their deductible has been met if they have some form of health insurance [30]. This review considered both direct and indirect inpatient OOP payments. Direct OOP cost was defined as payments made by patients for medical or healthcare services during an inpatient admission [31]. Indirect cost was defined as non-medical costs, such as income loss, transportation, meals, and accommodation paid by the patient during their hospitalisation [31].

Healthcare quality outcomes investigated were care complications, unplanned readmissions within 28 days, and unintended intensive care unit (ICU) admissions. Health outcomes investigated were quality of life, function, disability, in-hospital death, and prolonged hospital length of stay (LOS). Prolonged hospital LOS was defined as a hospital stay longer than the 75th percentile of the entire sample [32].

Studies were selected for inclusion if they were published in peer-reviewed journals, since 2000 and in English. Studies that examined OOP cost and quality in primary care settings, prescription-related OOP costs or OOP costs and treatment adherence were excluded. In addition, non-peer-reviewed articles, commentaries, case reports and conference abstracts were also excluded.

### Data sources and search strategy

A systematic search of the academic literature was conducted between January 2000 to October 2022 using five electronic databases, namely Scopus, Medline, Psych Info, CINAHL and Embase. The search strategy was developed using the key concepts of OOP costs and healthcare quality and outcomes by developing keywords, synonyms and phrases. An initial search was carried out in Medline to identify all the possible synonyms of the main concepts included in the study. Table 1 presents the search strategy applied to each database. Snowballing techniques to improve search sensitivity, such as reference list follow up and searching the contents of the relevant journals such as *BMJ Quality & Safety*, were applied. Endnote reference management software (Endnote 20.2.1) was used for storing extracted studies and Covidence systematic review software was used for data screening.

**Table 1:** Search terms and search strategy for use in the review

1	Intervention	out-of-pocket cost^a^ OR direct expenditure^a^ OR health expenditure^a^ OR out-of-pocket expenditure^a^ OR out-of-pocket expense^a^ OR out of pocket cost^a^ OR out of pocket expenditure^a^ OR out of pocket expense^a^ OR out-of-pocket payment^a^ OR out-of-pocket spending^a^ OR out of pocket payment^a^ OR out of pocket spending^a^ OR deductible^a^ OR co-pay OR co pay OR copay OR pocket cost^a^ OR patient spending^a^ OR patient cost^a^ OR co-insurance OR coinsurance OR health adj3 expenditure^a^ OR health adj3 spending^a^ OR gap payment OR cost sharing OR out-of-pocket fee^a^ OR out of pocket fee OR non-reimbursed cost^a^ OR prescription drug cost^a^ OR medical bill^a^
2	Outcome	quality OR outcome^a^ OR outcome assessment^a^ OR quality of life OR QOL OR surgical infection OR complication^a^ OR morbidity OR mortality OR death^a^ OR survival^a^ OR readmission^a^ OR patient experience^a^ OR patient satisfaction OR disability OR disabilit^a^ OR function OR ICU admission^a^ OR error^a^ OR length of stay OR LOS OR safety OR postoperative complications OR recovery time OR health status
**1 & 2**		**Combined using AND with the limit human, English and timespan limit between Jan 2000 to current.**

^a^Truncation symbol used to capture all possible variations of the word

### Study selection and data extraction

**Fig. 1:**
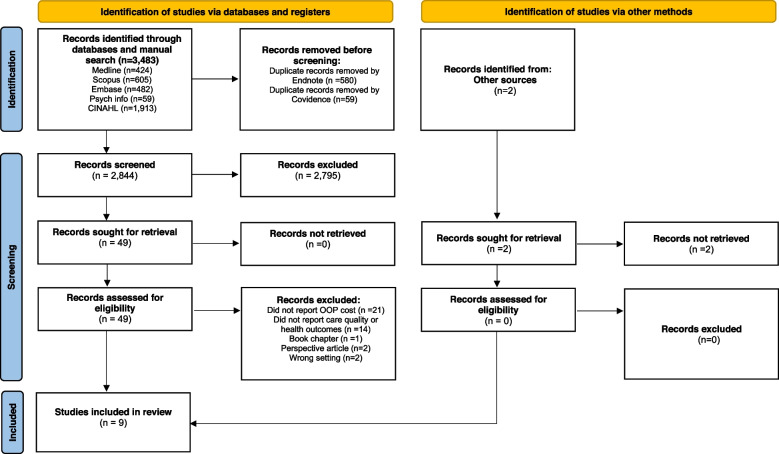
PRISMA flow diagram^1^ depicting literature search process. ^1^ From: *Page* et al. The PRISMA 2020 statement: an updated guideline for reporting systematic reviews. BMJ 2021;372: n71. https://doi.org/10.1136/bmj.n71

A three-step study selection process was employed, with three independent reviewers (RW, JL, SS) involved in all the three stages. In the first stage, 3,483 identified studies were exported into Endnote and 639 duplicates were removed. In the second stage, titles and abstracts of the remaining 2,844 articles were reviewed for their eligibility for inclusion. Those studies considered not to be relevant were excluded (*n* = 2,795). In the third stage, 49 eligible articles were independently examined in full for their inclusion. Final agreement was made by consensus between the three reviewers. Any discrepancy between the reviewers was discussed and settled by the two further team members (RH, RM). RH and RM conducted a face validity check on the final set of included articles (Fig. 1).

Data extraction was conducted by RW and verified by RH and RM. Information extracted included: authors, publication year, country of origin, study type, population, OOP cost type and inclusions, factors explored as determinants of OOP costs, healthcare outcomes assessed and major findings. The methodological quality of included studies was assessed using the CASP cohort checklist or CASP Randomised Control Trial (RCT) checklist [33, 34]. These frameworks appraise cohort studies across 12 domains and RCT studies across 11 domains and include a series of questions that can be answered with a yes/no/cannot tell response. To compare studies more effectively, studies scoring positively on more than 50% of the items were considered acceptable or of good quality in concordance with previous reviews [35, 36]. Studies with lower than 50% ‘yes’ responses were considered to be associated with bias. Two of the studies were quasi-experimental difference in difference studies and were appraised using the CASP cohort checklist as it was more appropriate than the RCT checklist.

### Data synthesis

The information on the included articles in the data table were compared using a narrative empirical synthesis as a quantitative synthesis was deemed inappropriate due to the heterogeneity in the study design, population, OOP cost measurements and the outcome measures investigated. The narrative synthesis involved discussing the tabulated data by the team members and identifying the patterns of associations and consistent findings in terms of study objectives. Further exploration aimed to identify the relationships between study characteristics and findings, and the influence of different OOP measures and outcome measures and study settings on the findings.

## Results

### Study characteristics

A total of nine studies were included in the review (Table 2). Three were conducted in the United States of America [37–39], two in Finland [40, 41], China [42, 43] and India [44, 45]. Five of the nine studies used a retrospective cohort design [41–45], two were quasi-experimental difference-in-difference studies [38, 39] and two were prospective cohort studies [37, 40], one of which had a RCT design [37]. Sample sizes varied from 152 to 423,634 individuals. While most studies were conducted among adults aged 18 years or older, two studies recruited only adults aged 65 years and older. Most studies focused on a specific condition or procedure; people with a diagnosis of cancer (2 studies), liver disease (1 study), chronic kidney disease (1 study), myocardial infarction (1 study), those scheduled for hip arthroplasty (1 study), and those who gave birth within the last 48 h (1 study).

**Table 2:** Characteristics of included studies

**Author**	**Year**	**Country**	**Study objective**	**Study type**	**Population, age, sample size**	**OOP costs type and inclusions**	**Factors explored as determinants of OOP costs**	**Healthcare outcomes assessed**	**Major findings**
Chen et al. [43]	2017	China	To examine cancer-related financial burden and its association with health-related quality of life (HRQoL) in lung cancer patients	Retrospective cohort study	*n* = 227 Individuals with lung cancer aged > 18 years	Direct medical costs, indirect medical costs, healthcare cost to income ratio	NA	HRQoL assessed using the Functional Assessment of Cancer Therapy-Lung (FACT-L) scale	No association between direct and indirect healthcare costs and HRQoL. Healthcare costs exceeding total annual household income was found to be associated with poorer HRQoL.
Choudhry et al. [37]	2014	United States (US)	To evaluate impact of full coverage (lower co-payments) on vascular events following myocardial infraction and its differential effect based on ethnicity	Randomised control trial	*n* = 2,387 Individuals < 65 years who had myocardial infraction	Individual’s OOP costs from insurers’ claims data	NA	Readmission to hospital	Full coverage (and therefore lower co-payments) was significantly associated with reduced rates of readmission in non-Caucasian people. No association was identified between level of coverage and readmission rates in the Caucasian population
Koskinen et al. [41]	2019	Finland	To examine the relationship between OOP costs and HRQoL among breast, prostate and colorectal cancer patients	Retrospective cohort study	*n* = 1,978 Individuals with breast, prostate or colorectal cancer aged ≥ 18 years.	Total OOP costs incurred including overheads, equipment and drugs for inpatient use, cancer medicines, specialist use and travel costs, if applicable	Stage of treatment and reported financial difficulty	HRQoL measured by two generic instruments, the 15D and the EQ-5D-3L, and by the cancer-specific EORTC-QLQ-C30 instrument.	Higher OOP costs were associated with poorer HRQoL. OOP payments were higher among palliative care patients and among individuals who self-reported higher health-related financial difficulties.
Landrian et al. [44]	2020	India	To investigate the association between total OOP expenditures and the quality of care and maternal complications following delivery	Retrospective cohort study	*n* = 2,018 Individuals who delivered within the last 48 h aged ≥ 18 years.	Total OOP costs calculated by summing the reported amounts paid by patients for transportation, labour and delivery care, medicines, medical tests and tips.	Age, education, occupation, place of residence, religion, caste, monthly income, wealth quintile, parity, level of facility, received Janani Suraksha Yojana programme benefits, number of health checks performed upon facility arrival, number of health checks performed during labour and delivery, number of health checks performed after delivery, experienced complications during or after labour and delivery	Quality of care (assessed by number of health checks performed) and experience of complications during or after labour and delivery	No association between OOP costs and any labour- or delivery-related quality of care indicators. OOP costs were highest among individuals who were younger, who had college education or higher, and greater wealth. Higher OOPs were experienced by women residing in urban areas (as opposed to rural), who delivered in district hospitals (as opposed to primary health clinic, community health centre and first referral unit) and among women who experienced complications. OOP costs decreased with the number of health checks. No differences in mean OOP costs were identified by occupation, religion or caste.
McHugh et al. [38]	2019	US	To examine the impact of increased levels of cost sharing on the use of hospital care and length of stay	Quasi-experimental difference-in-differences study	*n* = 423,634 Individuals enrolled in Medicare action plan aged ≥ 65 years	Change in OOP costs payment plans from deductible at admission to a daily co-payment method.	NA	Length of stay in hospital	No significant association observed between OOP costs and length of stay.
Montin et al. [40]	2007	Finland	To examine the relationship between OOP costs and HRQoL	Prospective cohort study	*n* = 100 Individuals scheduled for primary or revision hip arthroplasties. Mean age 63.9 years	OOP cost information over 6 months recorded by patients using a cost diary. Cost included hospital costs, outpatient department fee, rehabilitation unit fee, cost of pain killers bought after discharge, home nursing fee, physiotherapy costs and other possible costs such as help service fees, and transportation costs.	Age, time on waiting list, smoking, discharge to home	HRQoL measured post-operatively at 3 and 6 months compared with the preoperative level by using the Sickness Impact Profile (SIP)	No significant relationship observed between OOP cost and HRQoL OOP costs were greater among older patients. Lower OOP costs were associated with longer time on waitlist, smoking and discharge to home as opposed to discharge to other services.
Siddiqui et al. [39]	2015	US	To investigate whether emergency department (ED) co-payment enforcement was associated with increased inpatient length of stay	Quasi-experimental difference-in-difference study	n treatment = 3,122 n control = 7,433 Individuals enrolled in Medicaid aged 19 – 64 years	Enforcement of ED OOP cost payments	NA	Length of stay in hospital	No significant change reported in inpatient length of stay with OOP cost enforcement.
Valsa et al. [45]	2022	India	To whether the ratio of OOP payments to household income can affect HRQoL	Retrospective cohort study	*n* = 152 Individuals with chronic kidney disease undergoing haemodialysis aged 18 – 80 years.	Direct medical costs, indirect medical costs, healthcare cost to income ratio	Three dialysis per week Vs two per week and gender	HRQoL measured using EQ_5D instrument.	No association between direct and indirect OOP costs and HRQoL. Proportion of household income spend as OOP was found to be negatively associated with HRQoL. OOP costs were higher for patients on 3 dialysis per week compared to two per week. No difference in OOP between two genders
Xu et al. [42]	2017	China	To study the impact of patient cost sharing on use of treatments and procedures, mortality, hospitalisation cost and medication cost.	Retrospective cohort study	*n* = 10,858 Individuals with common liver diseases aged ≥ 18 years.	OOP measured by reimbursement ratio (% of reimbursed cost to total treatment cost). Higher reimbursement ratio denotes lower OOP	NA	Length of stay in hospital, in-hospital mortality	Lower OOP costs associated with longer inpatient length of stay. No significant association between OOP costs and inpatient mortality.

*NA* Not applicable

### Study quality

Additional file 1: Tables S1 and S2 provide the findings of the quality appraisal for each of the studies. Overall, all of the studies were considered to be of relatively good quality (> 50% ‘yes’ responses to the criteria) with a clear objective, appropriate outcome measurement, sound study design and acceptable recruitment processes. Exposure and outcome were self-reported in only one study [44]. Possible confounding factors were identified and adjusted in all studies except one [40]. A common limitation was the lack of representativeness of the sample to the general population, which created challenges for comparison and generalisation.

### Associations between OOP costs and quality and health outcomes

A variety of quality and health outcomes were explored in the included studies in a range of healthcare contexts. The small number of studies and heterogeneity in the patient populations and outcomes explored created challenges for drawing out common findings between the studies, but several associations between OOP cost and quality were identified. The review identified fourteen associations between OOP costs, healthcare quality and patient health outcomes from the nine studies. These included associations examining multiple measures of OOP costs, quality of care as well as the differential analyses. Nearly two thirds (9/14) did not identify a significant relationship between OOP costs and healthcare-related quality and outcomes.

Four studies examined the association between OOP costs and health-related quality of life (HRQoL) [40, 41, 43, 45]. Chen et al. [43] studied the association of OOP costs on HRQoL of patients with early-stage lung cancer admitted to the inpatient unit of an internal medicine-chest oncology hospital and found no association between direct and indirect OOP costs and HRQoL. Yet, a negative association was determined in the same study, between OOP cost and HRQoL for patients whose healthcare costs exceeded total annual household income. Similar findings of negative association between the proportion of household income spend as out of pocket payments and HRQoL and no association between direct and indirect OOP costs and HRQoL were reported in patients with kidney disease admitted for haemodialysis in a private hospital in India [45]. A negative association between OOP cost and HRQoL was also reported by Koskinen [41] for individuals with breast, prostate and colorectal cancer who represented all stages of disease from diagnosis to end of life care. Montin et al. [40] examined HRQoL in individuals who had hip arthroplasties in Finland and found no statistically significant association between OOP and HRQoL.

Three included studies reported on associations between OOP costs and hospital LOS [38, 39, 42]. McHugh’s [38] quasi-experimental study examining the impact of increased levels of cost sharing (in which patients contribute to service costs via OOP payments) on hospital LOS among older individuals (≥ 65 years), indicated no significant association of OOP costs on hospital LOS. Similarly, Siddique et al. [39], using a quasi-experimental study design, observed no association between OOP cost and hospital LOS following the enforcement of emergency OOP payments. However, a study from China examining the association between OOP costs and hospital LOS among individuals with liver diseases reported that lower OOP costs were associated with a longer inpatient LOS or poorer quality of care [42].

**Fig. 2:**
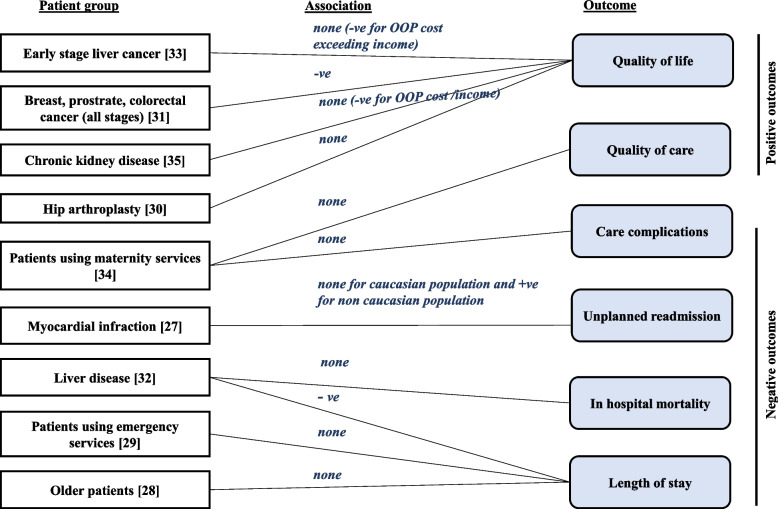
Summary of review findings

Outcomes beyond length of stay that captured quality of care were examined in three studies. One study examined quality of care determined by the number of health checks performed and care complications experienced following birth [44], another by readmission rates [37] and the third by in-hospital mortality [42]. The first of these studies demonstrated that total OOP cost was not associated with maternal quality of care following birth, assessed in terms of number of health checks performed and care complications [44]. Choudhry et al. conducted an RCT study in United States of America to evaluate the impact of full treatment cost coverage or lower co-payments on readmission rates following myocardial infraction and its differential effect based on ethnicity. Lower OOP payments were reported to be significantly associated with lower rates of readmission in non-Caucasian people, but not in the Caucasian population [37]. Xu et al. examined the association between OOP costs and inpatient mortality in individuals with liver diseases and reported no association [42]. The review findings are summarised as Fig. 2.

### Population and contextual determinants of OOP costs

Determinants of OOP costs were examined in four studies [40, 41, 44, 45]. Age was examined in two of the four studies [40, 44]. Other variables differed between the studies but included gender, education, occupation, place of residence, religion, caste, monthly income, parity, level of facility, number of health checks performed, complications during labour, stage of treatment, time on waitlist, smoking status, discharge to home as opposed to other services and amount of dialysis required per week.

OOP costs were reported to be higher among individuals who were young adults (18 – 24 years) and also those who had higher monthly income (US $150 or higher) among patients using maternity services [44]. Conversely, higher OOP costs were reported among older patients (mean age 75.1, SD 8.2 years) who underwent hip arthroplasty compared to their younger counterparts (mean age 62.1, SD 11.1 years) [40]. Greater self-reported financial difficulty (which was associated with employment status – employed or unemployed) was reported to be associated with higher OOP costs in individuals with breast, prostate and colorectal cancer by Koskinen et al. [41].

Individual studies reported OOP costs as higher among individuals who had a higher level of education (secondary, vocational, college or higher as opposed to none), who lived in urban areas as opposed to rural, by the level and type of health facility used (district hospitals as opposed to primary health clinic, community health centre and first referral unit) and by patients requiring more treatment numbers (three dialysis per week as opposed to two per week) [44, 45]. Stage of treatment (palliative care as opposed to other stages of cancer), complications experienced during care and discharge to services other than home were also reported to be associated with higher OOP costs [40, 41]. One study also reported that a shorter time on the surgical wait list and non-smoking were associated with higher OOP costs. However, these individuals were also those who were younger with less service use [40].

## Discussion

Health consumers around the world are increasingly seeking evidence to inform their decision-making regarding healthcare provider selection based on cost and quality information [46, 47]. Improving transparency on healthcare cost and quality of care is considered important for reducing the burden of OOP costs, so that consumers can make informed choices regarding their care [48]. Yet, internationally consumers report challenges in accessing information about the level of OOP costs they might face and whether higher OOP costs mean they are more likely to receive better quality of care or have enhanced health outcomes [49]. Efforts have already been made by many governments to pursue consumer price transparency. One such example is the Australian government’s *Medical Costs Finder* website that lists the median OOP costs for a selected specialities, services and regions [50]. Similarly, the *hospital price transparency* policy in the US requires hospitals to disclose all payer specific negotiated rates for all items and services in a consumer-friendly manner [51]. Private health insurance providers are also ramping up their efforts to improve price transparency [48]. However, a better understanding of the effect of higher OOP costs on the quality of care is vital to supplement these efforts so that consumers are able to make informed choices about their care.

The current review identified nine eligible studies, indicating a paucity of evidence. The studies identified were heterogenous in terms of the population, health systems and outcomes investigated. Despite the critical need for evidence on the nature of the relationship between OOP cost and quality of care, the scant evidence available and substantial heterogeneity creates challenges in establishing the nature of any association between OOP costs and healthcare quality and outcomes. Nonetheless, the most consistent finding was no significant association between OOP cost and inpatient quality of care (nine out of fourteen associations). A previous systematic review looking at hospital cost or hospital price which focussed only on the quality of inpatient care reported no general relationship between hospital cost/price and quality [19].

A negative association of OOP costs on patient health outcomes (i.e., higher OOP costs leading to poorer outcomes) were notable in studies exploring HRQoL in cancer and chronic kidney disease. In the context of cancer and chronic conditions, advanced stages, greater complexity and care requirements that are beyond the scope of private health fund allocation, could potentially be the contributing factors. Another potential explanation could be the association between financial burden caused by higher OOP costs imposed by these conditions and HRQoL [52–54].

Longer LOS in hospital is often considered an indicator of poorer quality of care and health outcomes [55, 56]. Yet evidence from multiple countries indicates that differences in LOS are often due to a range of factors including care complexity, but also an individual’s insurance status [57–59]. Connected to this is our findings from one included study that reported an association between lower OOP costs (generous reimbursement) and longer hospital LOS in China [42]. China’s current healthcare policy has been reported previously to provide perverse incentives to clinicians and hospitals to make healthcare decisions based not only on individuals’ health status but also on their insurance status [59].

The current review identified limited evidence in relation to the determinants of OOP costs and the quality of care. Age was the only variable explored across more than one study. Diverse study populations and clinical conditions demonstrated mixed findings with regards to age. OOP costs were reported to be higher in younger individuals accessing maternity services and among older patients who underwent elective hip arthroplasty procedure. A mixed association of age with catastrophic OOP costs depending on the clinical condition and complication levels were also reported by a previous systematic review looking at factors associated with the burden of household healthcare expenditures in Ethiopia [60].

Health care delivery systems across the world are structured and financed differently and this may have an impact on the OOP cost-quality association. The degree of regulation between public and private systems is one of the main determinants of OOP costs and may have implications for health care quality. For example, in the US, lacking universal health insurance, multimorbid patients are reported to spend less time in hospital and are discharged quickly to rehabilitative facilities leading to longer combined LOS across acute and non-acute facilities compared to countries with more generous long‐term hospital coverage such as Canada, Sweden, and Spain [61]. Similarly, largely publicly funded health systems, such as those of Finland, England and Australia, control the utilisation of specialist or hospital services by requiring a referral from primary care. However, this is not the case in US, Canada and India where direct access to specialist and hospital services are possible leading to patients paying higher costs for similar care [61–63]. These differences contribute to complex relationships between OOP costs and quality that are challenging to delineate in multi-country studies. The influence of different health service delivery models is therefore an important consideration.

### Strengths and limitations

This review is one of the first to synthesise information from the existing evidence to explore the association between OOP costs and healthcare quality and patient health outcomes in inpatient settings. Strengths include a systematic search strategy involving multiple data sources and reporting according to PRISMA guidelines. The findings have limitations including the low number of eligible studies available. A quantitative synthesis was not possible for this review due to the heterogeneity of the included studies. It is possible that some relevant studies were not captured by the search strategy. There remains a possibility of publication bias because non-English publications were omitted, and the grey literature was not searched. In addition, the focus of the review, specifically examining studies looking at the association between OOP cost and quality of inpatient care, could have resulted in not capturing studies examining general determinants of OOP costs.

## Conclusions and recommendations

This review suggests no beneficial effects of higher OOP costs on inpatient quality of care and health outcomes. The review provides indicative evidence that the association of OOP costs and healthcare quality is likely to be influenced by the health conditions examined, the population case-mix and the healthcare context, particularly regarding an individual’s health insurance status. The different healthcare policies and health system funding models may have an impact on the OOP cost-quality association. There is also potential to further explore the determinants of OOP costs to aid in identifying populations who face the greatest OOP costs and focus research and policy action on these groups to reduce the financial burden imposed by high OOP costs.

### Supplementary Information

**Additional file 1: Table S1.** Quality assessment of selected studies using CASP cohort checklist^a^. **Table S2.** Quality assessment of selected studies using CASP RAT checklist^b^.

## Data Availability

The data used and/or analysed during the current study will be available from the corresponding author on request.
