# Supplementary material for: Is there an association between out-of-pocket hospital costs, quality and care outcomes? A systematic review of contemporary evidence
Source: BMC Health Serv Res. 2023 Sep 13;23:984. doi: 10.1186/s12913-023-09941-3 (PMC10500869; doi:10.1186/s12913-023-09941-3)
Supplement: Supplementary file 1 — Additional file 1: Table S1. Quality assessment of selected studies using CASP cohort checklista. Table S2. Quality assessment of selected studies using CASP RAT checklistb. [file 12913_2023_9941_MOESM1_ESM.docx]

**Is there an association between out-of-pocket hospital costs, quality and care outcomes? A systematic review of contemporary evidence – Supplementary material**

**Table S1: Quality assessment of selected studies using CASP cohort checklist ^a^**

| **Authors** | **Q1** | **Q2** | **Q3** | **Q4** | **Q5a** | **Q5b** | **Q6a** | **Q6b** | **Q7** | **Q8** | **Q9** | **Q10** | **Q11** | **Q12** |
| --- | --- | --- | --- | --- | --- | --- | --- | --- | --- | --- | --- | --- | --- | --- |
| Chen et al [43] | Y | Y | Y | Y | Y | Y | NA | NA | No association between OOP costs and HRQoL. OOP cost exceeding income associated with poorer HRQoL | C | Y | Y | Y | Y |
| Koskinen et al [41] | Y | Y | Y | Y | Y | Y | NA | NA | Negative association between OOP costs and HRQoL | C | Y | Y | N | Y |
| Landrian et al [44] | Y | Y | N | N | Y | Y | NA | NA | No association between OOP costs and any labour and delivery-related quality of care | Y | Y | Y | Y | Y |
| McHugh et al [38] | Y | Y | Y | Y | N | Y | Y | Y | No association between change in OOP costs and length of stay | Y | Y | Y | Y | Y |
| Montin et al [40] | Y | Y | Y | Y | N | N | Y | Y | No significant relationship between cost and HRQoL. | C | Y | Y | Y | Y |
| Siddiqui et al [39] | Y | Y | Y | Y | Y | Y | Y | Y | No significant association between OOP costs and inpatient length of stay | C | Y | Y | Y | Y |
| Valsa et al [45] | Y | Y | Y | Y | N | Y | NA | NA | No association between OOP costs and HRQoL. OOP cost exceeding income associated with poorer HRQoL | C | Y | Y | Y | Y |
| Xu et al [42] | Y | Y | Y | Y | Y | Y | NA | NA | Lower OOP costs associated with longer inpatient length of stay. No significant association with inpatient mortality. | C | Y | Y | C | Y |

Y – Yes; N- No; C- Can’t tell; NA - Not applicable

^a^ CASP Cohort Appraisal Checklist questions:

| 1. Did the study address a clearly focussed issue? | 7. What are the result of this study? |
| --- | --- |
| 2. Was the cohort recruited in an acceptable way? | 8. How precise are the results? |
| 3. Was the exposure accurately measured to minimise bias? | 9. Do you believe the results? |
| 4. Was the outcome accurately measured to minimise bias? | 10. Can the results be applied to the local population? |
| 5a. Have the authors identified all important confounding factors? | 11. Do the results of this study ft with other available evidence? |
| 5b. Have they taken into account of the confounding factors in the design and/or analysis? | 12. Does the study have implications for practice? |
| 6a. Was the follow up of the subjects complete enough? |  |
| 6b. Was the follow up of subjects long enough? |  |

**Table S2: Quality assessment of selected studies using CASP RAT checklist ^b^**

| Authors | Q1 | Q2 | Q3 | Q4a | Q4b | Q4c | Q5 | Q6 | Q7 | Q8 | Q9 | Q10 | Q11 |
| --- | --- | --- | --- | --- | --- | --- | --- | --- | --- | --- | --- | --- | --- |
| Choudary et al | Y | Y | Y | N | C | C | C | Y | Y | Y | Y | Y | Y |

Y – Yes; N- No; C- Can’t tell; N/A - Not applicable

^b^ CASP RAT Appraisal Checklist questions:

| 1. Did the study address a clearly focused research question? | 7. Were the effects of intervention reported comprehensively? |
| --- | --- |
| 2. Was the assignment of participants to interventions randomised? | 8. Was the precision of the estimate of the intervention or treatment effect reported? |
| 3. Were all participants who entered the study accounted for at its conclusion? | 9. Do the benefits of the experimental intervention outweigh the harms and costs? |
| 4a. Were the participants ‘blind’ to intervention they were given? | 10. Can the results be applied to your local population/in your context? |
| 4b. Were the investigators ‘blind’ to the intervention they were giving to participants? | 11. Would the experimental intervention provide greater value to the people in your care than any of the existing interventions? |
| 4c. Were the people assessing/analysing outcome/s ‘blinded’? |  |
| 5. Were the study groups similar at the start of the randomised controlled trial? |  |
| 6. Apart from the experimental intervention, did each study group receive the same level of care (that is, were they treated equally)? |  |
